# Minimal Conductance-Based Model of Auditory Coincidence Detector Neurons

**DOI:** 10.1371/journal.pone.0122796

**Published:** 2015-04-06

**Authors:** Go Ashida, Kazuo Funabiki, Jutta Kretzberg

**Affiliations:** 1 Cluster of Excellence "Hearing4all", Carl von Ossietzky University Oldenburg, Oldenburg, Germany; 2 Systems Biology, Osaka Bioscience Institute, Suita, Osaka, Japan; University Paris 6, FRANCE

## Abstract

Sound localization is a fundamental sensory function of a wide variety of animals. The interaural time difference (ITD), an important cue for sound localization, is computed in the auditory brainstem. In our previous modeling study, we introduced a two-compartment Hodgkin-Huxley type model to investigate how cellular and synaptic specializations may contribute to precise ITD computation of the barn owl's auditory coincidence detector neuron. Although our model successfully reproduced fundamental physiological properties observed *in vivo*, it was unsuitable for mathematical analyses and large scale simulations because of a number of nonlinear variables. In the present study, we reduce our former model into three types of conductance-based integrate-and-fire (IF) models. We test their electrophysiological properties using data from published *in vivo* and *in vitro* studies. Their robustness to parameter changes and computational efficiencies are also examined. Our numerical results suggest that the single-compartment active IF model is superior to other reduced models in terms of physiological reproducibility and computational performance. This model will allow future theoretical studies that use more rigorous mathematical analysis and network simulations.

## Introduction

Sound localization, or the ability to find the location of a sound source, is one of the fundamental functions of the auditory system. The interaural time difference (ITD), which is the difference in arrival timing of acoustic waves at the two ears, is an important cue for azimuthal sound localization (see [1–4] for reviews). In mammals, ITD is computed in the medial superior olive (MSO), where inputs from the bilateral cochlear nuclei converge [5–8]. In birds and reptiles, binaural neurons in the third order auditory station, nucleus laminaris (NL), show sensitivity to ITDs [9–12]. Recent advances in electrophysiological recording techniques *in vivo* allow us to examine how binaural synaptic inputs are computed in these neurons [13,14]. Since MSO and NL neurons sense precise timing of synaptic inputs to change their spike rates, they are often called auditory coincidence detectors.

Conductance-based Hodgkin-Huxley-type (HH) models have been widely used for investigating biophysical mechanisms of ITD coding in auditory coincidence detector neurons [15–21]. These modeling studies have revealed that finely-tuned synaptic and cellular properties are essential for precise ITD computation. The HH-type model is useful to study detailed biophysical effects of channel kinetics, but it requires a large computational power [22]. Furthermore, mathematically rigorous analysis of HH-type models is generally difficult due to the large number of parameters and nonlinearities. To circumvent these difficulties, more abstract integrate-and-fire (IF) models with fewer parameters were also used in previous theoretical studies of sound localization [19,23–25], although the biophysical basis of the IF model is much weaker than the HH model [22,26]. Combining the strengths of the both models, Svirskis and Rinzel [27] introduced an IF model with low-voltage-activated potassium (K_LVA_) conductance to study how K_LVA_ channels contribute in the detection of weak signals in the model MSO neuron.

In our previous studies, we used HH-type two-compartment models that mimicked physiological characteristics of the barn owl's NL neuron [13,28,29]. Although our models were able to explain the roles of various biophysical factors in ITD computation, it was still unclear which model features were essential for binaural sound information processing. Thus, in the present study, we reduce our two-compartment NL model into three simplified models and compare simulation results with available physiological data. The main goal of this study is to find a minimal NL neuron model that reproduces known fundamental electrophysiological properties related to ITD coding. Numerical efficiency and reliability of the models are also examined.

## Materials and Methods

### Model construction

**Fig 1:**
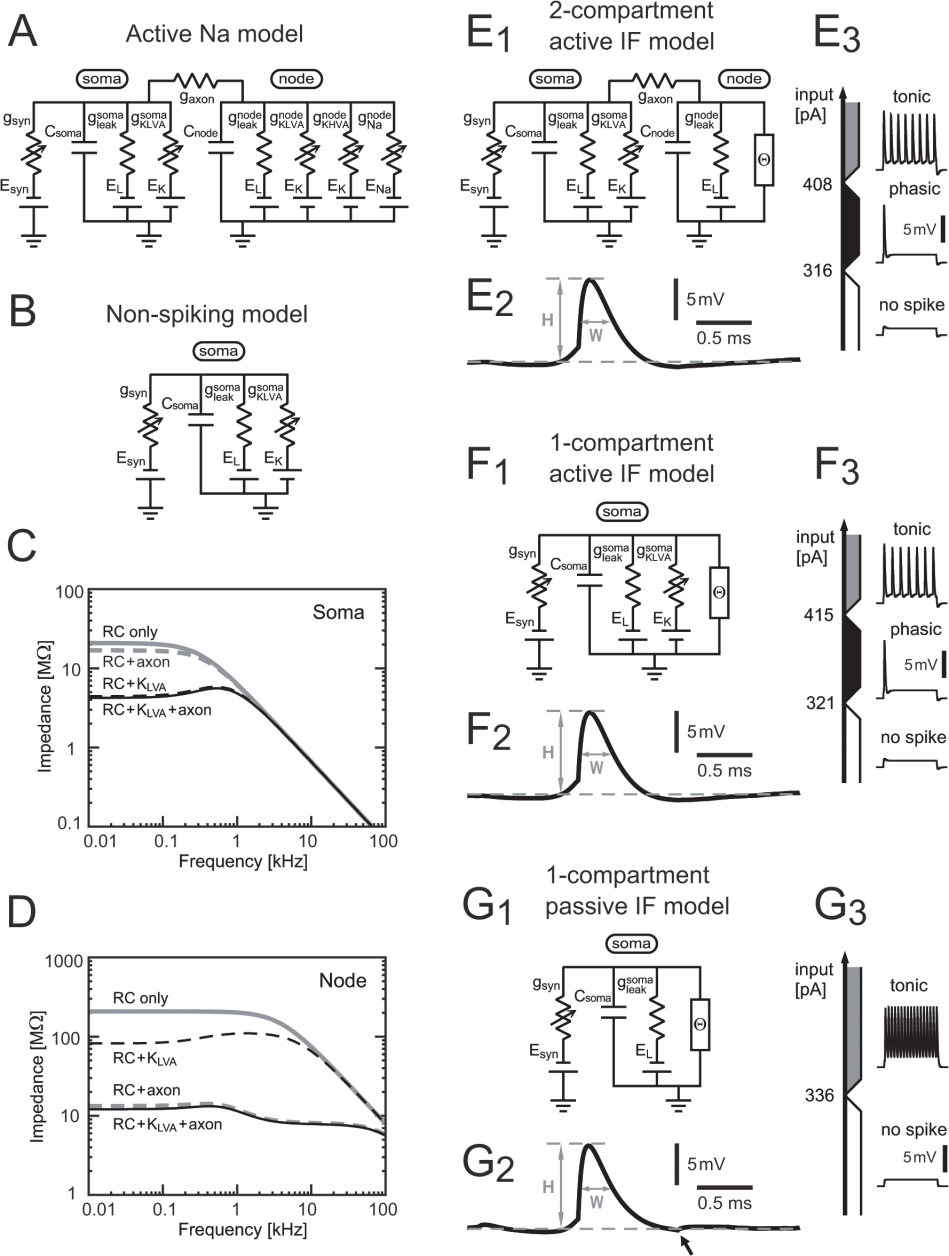
NL models. (A) Circuit of the conductance-based two-compartment HH-type "active Na" model [13,29]. The somatic compartment contains leak and K_LVA_ currents whereas the nodal compartment has high voltage activated potassium (K_HVA_) and Na currents (required for spike generation) in addition to leak and K_LVA_. (B) Circuit of the "non-spiking" conductance-based single-compartment model with leak and K_LVA_ currents. This model was designed for simulating subthreshold membrane responses [13,32]. (C) Membrane impedance of the somatic compartment of the active Na model. (D) Membrane impedance of the nodal compartment of the active Na model. (C-D) In the "RC only" and "RC + K_LVA_" conditions, *g*_axon_ was fixed to zero (i.e., no axonal current) to isolate the compartment. In the "RC only" and "RC + axon" conditions, *g*_KLVA_ of the compartment was fixed to zero. Membrane potential was fixed at -61 mV. (E_1-3_) Two-compartment active IF model: (E_1_) circuit diagram, (E_2_) average spike shape at the soma, and (E_3_) membrane responses to DC step current injection. Spike amplitude H = 9.7 mV. Half-amplitude spike width W = 0.3 ms. (F_1-3_) Single-compartment active IF model: (F_1_) circuit diagram, (F_2_) average spike shape, and (F_3_) membrane responses to DC step current injection. Spike amplitude H = 9.8 mV. Half-amplitude spike width W = 0.3 ms. (G_1-3_) Single-compartment active IF model: (G_1_) circuit diagram, (G_2_) average spike shape, and (G_3_) membrane responses to DC step current injection. Spike amplitude H = 9.8 mV. Half-amplitude spike width W = 0.3 ms. (E_1_, F_1_, G_1_) Θ denotes the IF spike generator. (E_3_, F_3_, G_3_) Holding potential = -60 mV.

Anatomical [30,31] and physiological [13] evidence show that spikes in an avian NL neuron are initiated at a remote site from the cell body, presumably at the first node of Ranvier. Our previous NL neuron model [28,29] consisted of two compartments (Fig 1A). The somatic compartment receives synaptic inputs, whereas the nodal compartment serves as a spike generator. We refer to this model as the **"Active Na"** model, because action potentials are generated by the Na conductance in the nodal compartment. In this study, we reduced the active Na model in three steps. First, active conductances in the nodal compartment were replaced with a thresholding unit (shown by Θ in Fig 1E_1_; see next section for equations). Active and passive properties of the soma remain unchanged between these two models. This reduced model was named **"Two-compartment active IF"** model. The word "active" indicates that the model reserves low-voltage-activated (K_LVA_) conductance in the soma.

As the second step, we replaced the entire nodal compartment and the axon with a single thresholding unit in the soma (Fig 1F_1_; see below for equations). This new model was labeled as **"Single-compartment active IF model"**. This model can also be regarded as a simple expansion of the **"Non-spiking"** NL model (Fig 1B), which was introduced in our previous research to study subthreshold membrane responses [13,32].

As the last step, we dropped the K_LVA_ conductance from the soma and obtained the **"Single-compartment passive IF"** model (Fig 1G_1_). Similar models to this passive IF model (with slightly different membrane parameters) were already used in previous theoretical studies of sound localization [23,24].

#### Two-compartment active IF model

The two-compartment active IF model consists of the cell body (soma) and the node, which are connected with an axonal resistance (Fig 1E_1_). The membrane potentials of the soma (*V*_soma_) and node (*V*_node_) obey the standard membrane equations:

$$C_{\text{soma}}\frac{\text{d}}{\text{dt}}V_{\text{soma}}(t)=I_{\text{leak}}^{\text{soma}}+I_{\text{KLVA}}+I_{\text{axon}}^{\text{soma}}+I_{\text{syn}}+I_{\text{ext}}$$

and

$$C_{\text{node}}\frac{\text{d}}{\text{dt}}V_{\text{node}}(t)=I_{\text{leak}}^{\text{node}}+I_{\text{axon}}^{\text{node}}+I_{\text{IF}},$$

where *C*_soma_ and *C*_node_ are the membrane capacitances,

$$I_{\text{leak}}^{\text{soma}}=g_{\text{leak}}^{\text{soma}}(E_{\text{L}}-V_{\text{soma}})\;\text{and}\;I_{\text{leak}}^{\text{node}}=g_{\text{leak}}^{\text{node}}(E_{\text{L}}-V_{\text{node}})$$

are the leak currents,

$$I_{\text{KLVA}}={\bar{g}}_{\text{KLVA}}\;d(V_{\text{soma}},t)\;(E_{\text{K}}-V_{\text{soma}})$$

is the K_LVA_ current,

$$I_{\text{axon}}^{\text{soma}}=-I_{\text{axon}}^{\text{node}}=g_{\text{axon}}(V_{\text{node}}-V_{\text{soma}})$$

is the axonal current,

$$I_{\text{syn}}=g_{\text{syn}}(E_{\text{syn}}-V_{\text{soma}})$$

is the synaptic input to the soma, *I*_IF_ is the current induced by the IF thresholding unit (defined below), and *I*_ext_ is the external stimulus current (set to zero by default). The activation variable *d*(*V*,*t*) of the K_LVA_ conductance in the soma obeys the first-order differential equation:

$$\frac{\text{d}}{\text{dt}}d(V,t)=\frac{d(V,t)-d_{\infty }(V)}{\tau_{d}(V)}.$$

The K_LVA_ kinetics was taken from a previous slice study [33]:

$$\tau_{d}(V)={\text{Q}}_{10}^{({\text{T}}_{1}-{\text{T}}_{0})/10}/(\alpha_{d}(V)+\beta_{d}(V)),$$

$$d_{\infty }(V)=\alpha_{d}(V)/(\alpha_{d}(V)+\beta_{d}(V)),$$

$$\alpha_{d}(V)=0.20\;\exp((V+60)/21.8),$$

$$\beta_{d}(V)=0.17\;\exp(-(V+60)/14),$$

with Q_10_ = 2.5, T_0_ = 23 ^o^C, T_1_ = 40 ^o^C. The unit for *α*_*d*_ and *β*_*d*_ is 1/ms.

Parameter values are the same as our previous active Na model [29] and summarized in Table 1. The K_LVA_ conductance in the soma was unchanged between the active Na model and the active IF model, because it plays an essential role in characterizing the membrane impedance (Fig 1C). The K_LVA_ conductance in the node, however, was eliminated from the present active IF model, because it has only limited effects on the nodal membrane impedance, which is dominated by the axonal conductance (Fig 1D).

**Table 1:** Default parameter values for the membrane dynamics of the three reduced models.

Parameter	2-comp active IF	1-comp active IF	1-comp passive IF
C_soma_	24 pF	24 pF	24 pF
$g_{\text{leak}}^{\text{soma}}$	48 nS	48 nS	240 nS
${\bar{g}}_{\text{KLVA}}$	192 nS	192 nS	—-
C_node_	0.2 pF	—-	—-
$g_{\text{leak}}^{\text{node}}$	2 nS	—-	—-
*g*_axon_	117.8 nS	—-	—-
*E*_syn_	0 mV	0 mV	0 mV
*E*_L_	-60 mV	-60 mV	-60 mV
*E*_K_	-75 mV	-75 mV	—-

These parameters were fixed throughout our simulations.

The current from the thresholding unit consists of two parts: *I*_IF_ = *I*_const_ + *I*_spike_. The first term corresponds to the constant opening of ion channels in the nodal compartment. The second term *I*_spike_ denotes the spike-associated transient current: when the nodal membrane potential *V*_node_ crosses the threshold V_θ_ at time *t* = T_θ_, a spike current *I*_spike_(*t*-T_θ_) is initiated. The spike current is modeled as a sum of two exponential functions:

$$I_{s\text{pike}}(t)={\text{A}}_{1}\text{exp}(-t{/\tau }_{1})+{\text{A}}_{2}\exp(-t{/\tau }_{2})\;\left(\text{for}\;t\geq 0\right),\;=\;0\;\left(\text{for}\;t<0\right).$$

We chose exponential functions because they enable exact calculation at discrete time steps [34]. Once the membrane potential reaches the threshold V_θ_, the threshold-crossing detector will be in an absolute refractory period of T_ref_. Namely, V_θ_ = ∞ for T_θ_ < *t* < T_θ_+T_ref_. See Table 2 for the default parameter values of the thresholding unit, which were fixed throughout our series of simulations unless otherwise stated.

**Table 2:** Default parameter values for the thresholding unit of the three reduced models.

Parameter	2-comp active IF	1-comp active IF	1-comp passive IF
*I*_const_	200 pA	200 pA	200 pA
A_1_	4000 pA	3500 pA	4000 pA
A_2_	3000 pA	3000 pA	4000 pA
τ_1_	0.02 ms	0.02 ms	0.02 ms
τ_2_	0.20 ms	0.20 ms	0.20 ms
V_θ_	-56.7 mV	-58.3 mV	-58.6 mV
T_ref_	0.9 ms	0.9 ms	0.9 ms

These parameters were fixed throughout our simulations unless otherwise stated.

#### Single-compartment active IF model

In the single-compartment IF model, the spike-initiating node of the two-compartment model together with the axonal resistance was reduced into a thresholding unit in the soma (Fig 1F_1_), while other ionic conductances of the soma were unchanged. The membrane potential of the soma (*V*_soma_) satisfies the equation:

$$C_{\text{soma}}\frac{\text{d}}{\text{dt}}V_{\text{soma}}(t)=I_{\text{leak}}^{\text{soma}}+I_{\text{KLVA}}+I_{\text{syn}}+I_{\text{IF}}+I_{\text{ext}}.$$

The current from the IF unit is the same as in the two compartment model: *I*_IF_ = *I*_const_ + *I*_spike_. The equations and parameter values for *C*_soma_, $I_{\text{leak}}^{\text{soma}}$, *I*_KLVA_, *I*_syn_, *I*_const_, and *I*_ext_ are the same as those in the two-compartment active IF model introduced above (see Table 1 for parameters). Thus the subthreshold membrane property is essentially identical to that of the two-compartment model.

The spike initiation mechanism is similarly defined. When the membrane potential *V*_soma_ reaches the threshold V_θ_ at time *t* = T_θ_, a spike current *I*_spike_(*t*-T_θ_) is injected. Note that, in the single-compartment model, spike currents are applied directly to the soma. The spike current *I*_spike_(*t*) is similarly defined as above with a set of slightly different parameters. The definition of the absolute refractory period T_ref_ was the same as that of the two-compartment model (see Table 2 for parameters).

#### Single-compartment passive IF model

By replacing the active K_LVA_ conductance with passive leak conductance, we obtain the single-compartment passive IF mode (Fig 1G_1_). The membrane potential of the soma (*V*_soma_) obeys the passive equation:

$$C_{\text{soma}}\frac{\text{d}}{\text{dt}}V_{\text{soma}}(t)=I_{\text{leak}}^{\text{soma}}+I_{\text{syn}}+I_{\text{IF}}+I_{\text{ext}}.$$

The equations and parameter values for *C*_soma_, $I_{\text{leak}}^{\text{soma}}$, *I*_syn_, *I*_const_, and *I*_ext_ are the same as those in the above-defined models except for the leak conductance $g_{\text{leak}}^{\text{soma}}$. The definitions for thresholding and spike initiation were also the same as in the single-compartment active IF model.

#### Membrane and threshold parameters

We used the same parameter sets for the soma of the active IF models (Table 1) as used for our previous active Na model [29]. With the parameters shown in Table 1, somatic membrane resistance at -61 mV was 4.4 MΩ (membrane time constant: ~0.1 ms), which was comparable to the electrophysiological data from the owl NL [13]. In the passive IF model, the somatic leak conductance was increased so as to keep the membrane resistance. Parameters for the spike-associated current (Table 2) were determined so that simulated spike waveforms resembled what was observed *in vivo* [13]. To date, there is no report available that systematically studied the voltage threshold V_θ_ and the refractory period T_ref_ of owl NL neurons. We determined the threshold of each model (Table 2) to make the "modulation depth" (defined later) of the simulated spike rates close to maximum. The refractory period was fixed to 0.9 ms. Possible effects of varying these parameters will be examined in Results.

#### Synaptic inputs

An NL neuron receives phase-locked inputs from converging axons of nucleus magnocellularis (NM) neurons on ipsi- and contralateral sides [9]. We used the same modeling procedure that was introduced in our previous study [11,32]. Briefly, phase-locked spikes of NM axons were modeled as an inhomogeneous Poisson process, whose intensity function λ(*t*) is periodic with the tonal stimulus frequency *f*_stim_. The degree of phase-locking was measured as vector strength [5], which was analytically related to the intensity function λ(*t*) [32]. Presynaptic spikes (modeled as delta functions) from each side were processed by a linear synaptic filter:

$$\alpha (t)={\text{H}}_{\alpha }(t{\text{/τ}}_{\alpha })\;\text{exp}(1-t{/\tau }_{\alpha })\;\left(t\geq 0\right),\;=0\;\left(t<0\right)$$

to obtain synaptic conductances *g*_ipsi_(*t*) and *g*_contra_(*t*). We assumed that all inputs from each side are locked to the same phase at the same frequency, and thus the ITD only affects the relative phase δ of the model inputs from the two sides:

$$g_{\text{syn}}(t)=g_{\text{ipsi}}(t)+g_{\text{contra}}(t+\delta /2\pi f_{\text{stim}}).$$

We used the same input parameters as in our previous modeling study [29,32]: *f*_stim_ = 4000 (Hz), average intensity λ_0_ = 500 Hz [35], vector strength r = 0.6 [36], number of NM inputs from each side M = 150 fibers [37], half amplitude width of the unitary synaptic input W_α_ (= 2.446 τ_α_) = 0.1 ms, amplitude of unitary synaptic input H_α_ = 1.3 nS. All synaptic inputs were assumed to converge onto the somatic compartment, because the owl's mid-to-high frequency NL neurons have only short and stubby dendrites [37].

### Model evaluation

**Fig 2:**
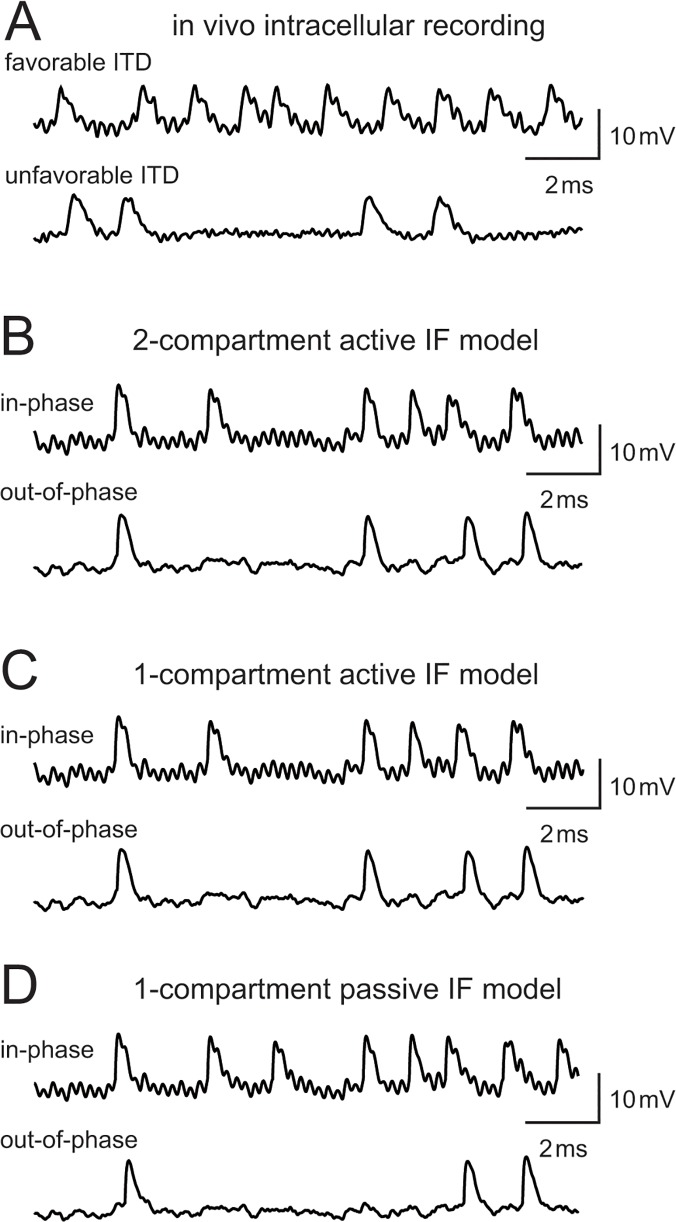
Membrane potential traces. (A) *In vivo* intracellular recording from a barn owl's NL neuron (data taken from [13]). Tonal stimulus at the best frequency (3600 Hz) was presented to the both ears by changing ITDs. (B) Simulated membrane potential of the two-compartment active IF model. (C) Simulated membrane potential of the single-compartment active IF model. (D) Simulated membrane potential of the single-compartment passive IF model. (B-D) Simulated binaural inputs are injected to the model neurons (see Materials and Methods for detail). "In-phase" means that the oscillation peaks of both inputs coincide resulted in the largest oscillation amplitudes. "Out-of-phase" means that the oscillating bilateral synaptic inputs are cancelled with each other to minimize the signal component at *f*_stim_ = 4000 Hz [29].

In order to evaluate the physiological reproducibility of the model neurons, we compare model outputs with published electrophysiological data. We examined spike waveforms, response types to step current injections, and spike rate modulations to simulated binaural inputs. To evaluate the numerical properties of the models, we examined their robustness to parameter changes and computational efficiency. For comparing membrane potential traces (Fig 2) we used the same data set from our previous *in vivo* intracellular recording study of the barn owl's NL [13].

#### Spike shape

For the calculation of average spike shapes (Fig 1E_2_, 1F_2_ and 1G_2_), we applied noise synaptic input to the model neurons. Random presynaptic inputs were modeled as non-phase-locked spike sequences (i.e., homogeneous Poisson process with an intensity λ_0_ of 500 Hz and a number M of 300 fibers), and were convolved with the synaptic filter *α*(*t*) to obtain a simulated synaptic input *g*_syn_(*t*). Spikes are aligned to the threshold crossing point and then averaged. 1500 spikes were estimated to obtain an average spike waveform. Data from our previous *in vivo* intracellular recording [13] were used as a reference (typical values: spike amplitude = 4–13 mV, spike width = 0.3–0.5 ms).

#### Response to step currents

In order to see the model membrane response to step current injection *I*_step_ (Fig 1E_3_, 1F_3_, and 1G_3_), we hold the membrane potential of each model at -60 mV by a constant current *I*_base_, and additionally applied step currents of varied amplitudes: *I*_ext_ = *I*_base_ + *I*_step_. The model response was classified as **"no spike"** when the current injection did not induce an action potential. The response was categorized as **"phasic spiking"** when only a single spike at the onset of the step current was observed. The response was referred to as **"tonic spiking"** when repetitive spikes occurred during the step current injection. We examined whether the model neurons show a phasic-spiking property as is typical in auditory coincidence detectors (e.g., [38]).

#### Responses to binaural inputs and rate-phase difference curve

**Fig 3:**
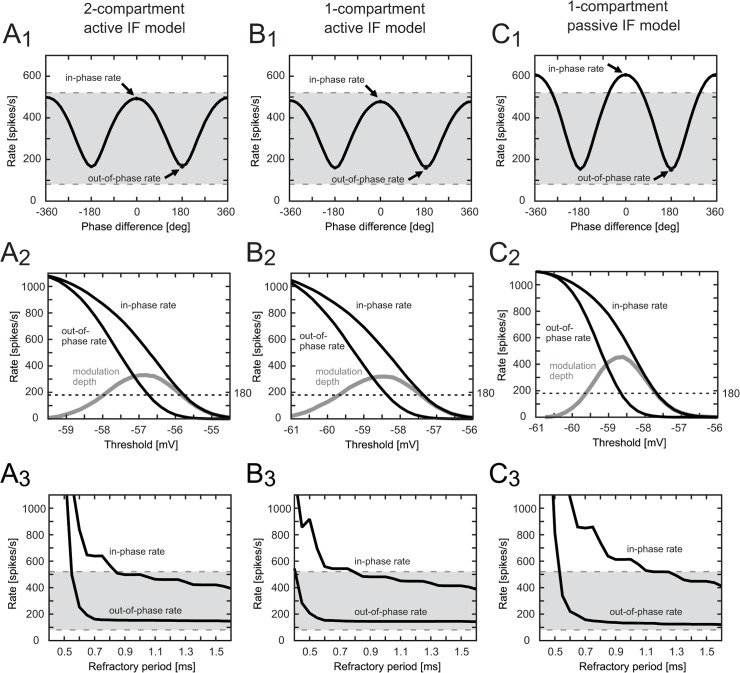
Spiking properties of the reduced models. (A_1-3_) Two-compartment active IF model. (B_1-3_) Single-compartment active IF model. (C_1-3_) Single-compartment passive IF model. First row (A_1_,B_1_,C_1_): simulated spike rates plotted against the phase difference δ of the bilateral inputs. Peaks appear at δ = 0 or ±2π (in-phase rate) whereas troughs appear at ±π (out-of-phase rate). Second row (A_2_,B_2_,C_2_): simulated spike rates plotted against the threshold of the IF unit. Refractory period was fixed to 0.9 (ms). Modulation depth (gray lines) is defined as the difference between the in-phase and out-of-phase rates (black lines). Dotted lines show the criterion of 180 spikes/sec for the modulation depth. Third row (A_3_,B_3_,C_3_): simulated spike rates plotted against the length of the refractory period. Threshold was fixed to (A_3_) -56.7, (B_3_) -58.3, and (C_3_) -58.6 mV, respectively. Shaded area of each panel in the first and third rows shows the range of typical discharge rates of the owl's NL neuron (see Materials and Methods for the definition).

We changed the phase difference δ of the bilateral model inputs (see above for definition) and calculated traces (Fig 2) and a rate-phase difference curve (Fig 3A_1_, 3B_1_, and 3C_1_) for each reduced model. The maximum spike rate is called the **"in-phase rate"**, because it is attained when δ was zero or integer multiples of 2π [29]. The minimum rate, obtained with δ = (2N+1)π (N = 0, ±1,…), was referred to as the **"out-of-phase"** rate. Note that the simulated in-phase rate corresponds to the spiking rate with a "favorable ITD" in physiological experiments, whereas the out-of-phase rate is comparable to the rate with an "unfavorable ITD" [13]. Discharge rates of owls' NL neurons for favorable and unfavorable ITDs were systematically measured by Peña et al. [35]. Based on their results, we defined the "typical discharge range" as 79–522 spikes/sec, which was derived from the "mean-SD" of the discharge rate for unfavorable ITD and "mean+SD" for favorable ITD. We used this typical discharge range as a criterion to evaluate the binaural responses of the models, and targeted to fit in this range when determining the thresholding parameters.

#### Robustness to parameter changes

To examine the robustness to changes in thresholding parameters, we changed the threshold V_θ_ and the refractory period T_ref_ of each IF model around the default values. We tested whether the in-phase and out-of-phase rates were within the typical discharge range. We additionally tested whether the "modulation depth" (defined as the difference between the in-phase and out-of-phase rates) exceeded our criterion of 180 spikes/sec, which is derived from the spike rate changes between favorable and unfavorable ITDs [35].

#### Numerical efficiency and reliability

**Fig 4:**
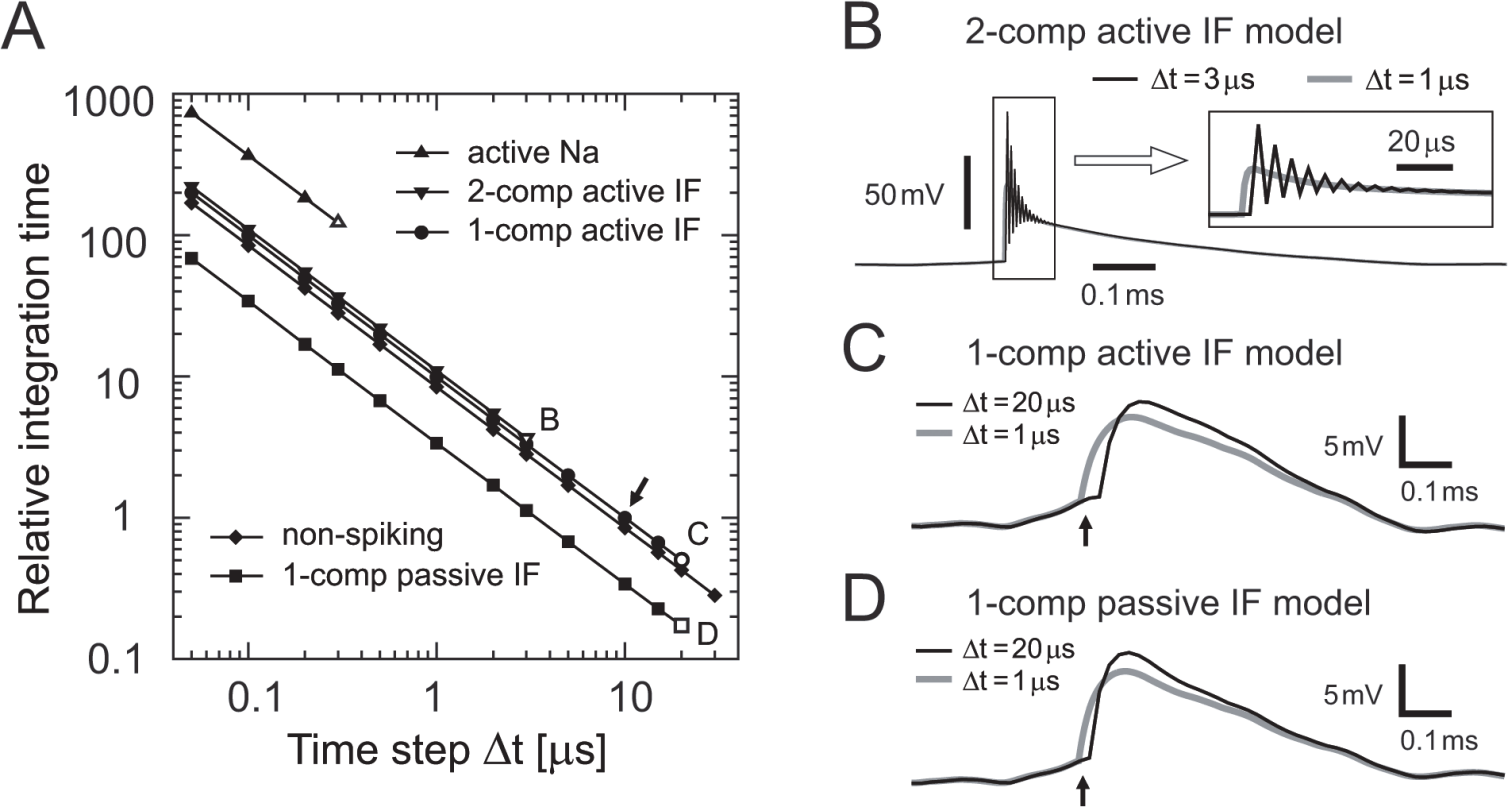
Model efficiency and reliability. (A) Relative integration time of the active Na (upward triangles), two-compartment active IF (downward triangles), single-compartment active IF (circles), non-spiking (diamonds), and single-compartment passive IF (squares) models. Single-compartment active IF model with Δt = 10.0 μs (small arrow) was used as the reference point (i.e., relative integration time = 1.0). Open symbols denote numerical unreliability with corresponding panels indicated by the small letters. (B) Unreliability of the two-compartment active IF model. A large time step (Δt = 3 μs, thin black line) leads to an erroneous oscillation in the nodal potential induced by spike currents. Inset shows a magnified view of the spike-induced unreliability, which does not occur with a sufficiently small time step (e.g., Δt = 1 μs, gray line). (C) Unreliability of the single-compartment active IF model. Too large a time step (Δt = 20 μs in this example) leads to an imprecise computation of the somatic shape. (D) Unreliability of the single-compartment passive IF model. Too large a time step (Δt = 20 μs in this example) leads to an imprecise computation of the spike shape. Small arrows in C and D show the timings when the simulated traces with Δt = 20 μs started to diverge from the traces with Δt = 1 μs by more than 0.5 mV.

As in our previous studies [28,29,32], we used the explicit (forward) Euler method for the numerical integration of the model equations. We varied the time step Δt from 0.1 μs to 30.0 μs. To examine the model efficiency, we calculated an average integration time of ten 1-second traces (i.e., 10 s in total) for each model. To test the numerical reliability, we compared simulated membrane potentials with different values of Δt. A numerical result was regarded as "unreliable" when the maximum discrepancy in the simulated potential differed by more than 0.5 mV from the potential calculated with the smallest time step of Δt = 0.1 μs. Note that this unreliability usually occurs at each spike initiation (see Fig 4). Numerical algorithms were implemented in D [39] and simulations were performed on a desktop computer (Dell Precision T1700) with 64 bit Windows 7 Professional Operating System, Intel Xeon CPU E3-1270 v3 (4 core, 3.5 GHz), and a 16 GB memory.

## Results

### Basic properties of reduced models

As introduced in Materials and Methods, we stepwise reduced the active Na model (Fig 1A) into three simpler models: the two-compartment active IF model (Fig 1E_1_), the single-compartment active IF model (Fig 1F_1_), and the single-compartment passive IF model (Fig 1G_1_). Using a similar approach as Svirskis and Rinzel [27], spike generating conductances in the active models were replaced by a threshold-crossing detector, while the K_LVA_ conductance of the cell body was kept intact. The values for K_LVA_ and leak conductances were chosen so that the membrane resistance matched previous *in vivo* measurements [13].

Spike shapes of the reduced models are shown in Fig 1E_2_, 1F_2_ and 1G_2_. We selected the parameters for spike-associated currents *I*_spike_ so that the simulated spike amplitude (9.7–9.8 mV) and half-peak width (0.3 ms) of all three models corresponded to *in vivo* recording data [13]. For the passive model, a small effect of the refractory period was seen in the spike trace (arrow in Fig 1G_2_), which was not evident for the active models.

Due to the large K_LVA_ conductance [40], the active models showed phasic spiking to step current injections (Fig 1E_3_ and 1F_3_), as was found in auditory brainstem neurons *in vitro* (chicken NL [41]; chicken NM [33,42]; mammalian MSO and octopus cells [38]). As shown previously [40,43], removing the K_LVA_ current resulted in the lack of phasic spiking mode with a direct transition from no spike to tonic spiking (Fig 1G_3_).

### Binaural phase-coding

Our previous *in vivo* intracellular recordings from owls NL [13] showed that phase-locked synaptic input induces an oscillating membrane potential. The oscillation amplitude depends on ITD (Fig 2A; see [13] for detailed discussion). Similar oscillations were found in the non-spiking model (Fig 1B; see [29,32] for theoretical examinations). All three reduced models, which have similar subthreshold response properties to the non-spiking model, also show these oscillations (Fig 2B–2D). The amplitude of the oscillation was maximal for δ = 0 (i.e., binaural inputs arrive in-phase) and minimal for δ = π (i.e., binaural inputs arrive out-of-phase) [29,32].

Spikes in auditory coincidence detector neurons are in general small at the cell body (gerbil MSO [44]; chicken NL [31]; owl NL [13]) probably because the actual spike initiation site is located away from the soma [28,30]. Reflecting this property, both measured (Fig 2A) and simulated (Fig 2B–2D) waveforms showed sinusoidal oscillations at the falling phase of spikes. It should be noted that, in the single compartment models, the spike current mimics the backpropagating spikes from the spike initiation site.

We changed the relative timing of ipsi- and contralateral inputs and calculated the spike rates of the model neurons. The maximum (in-phase) and minimum (out-of-phase) spike rates of the two active models were within the typical discharge rates of 79–522 spikes/sec (gray areas of Fig 3A_1_ and 3B_1_; see Materials and Methods for the definition) derived from previous owl NL recordings *in vivo* [35]. The single compartment passive IF model, however, showed a steeper rate-phase difference curve with the in-phase rate exceeding the typical range (Fig 3C_1_).

### Effect of parameter changes

In order to examine the parameter dependence of the model, we varied the parameter values of the thresholding unit (Fig 3A_2_, 3B_2_ and 3C_2_). First we varied the threshold V_θ_. Both the in-phase and out-of-phase rates decreased with increasing threshold (Fig 3A_2_ and 3B_2_, black lines). The spike modulation depth, which was defined as the difference between the in-phase and out-of-phase rates, showed a mild peak around the default threshold of the active models (Fig 3A_2_ and 3B_2_, gray lines). This qualitative tendency was observed in all the reduced models, but the single-compartment passive IF model was most strongly affected by threshold changes, showing steepest changes in the modulation depth (Fig 3C_2_). The range of thresholds for which the modulation depth exceeds our criterion of 180 spikes/sec was very similar for all the models (about 2.2 mV).

Next we changed the absolutely refractory period T_ref_ (Fig 3A_3_, 3B_3_ and 3C_3_). The length of the refractory period generally affects the maximum spike rate of an IF model (e.g., [45]). Since owls' NL neurons sometimes show discharge rates exceeding 600 spikes/sec [9] or even higher [13], we restricted the value of T_ref_ below 1.6 ms. When the refractory period exceeded 0.8 ms, it affected only moderately the in-phase-rate of the two-compartment IF model and almost negligibly the out-of-rate rate (Fig 3A_3_). If the period was shortened below 0.6 ms, both rates rapidly blew up to an unreasonable range. The refractory effect was even milder for the single-compartment active model (Fig 3B_3_). The out-of-phase spike rate of the passive model, however, was more sensitive to refractory periods than the other two active models (Fig 3C_3_). For example, varying T_ref_ from 0.8 to 1.6 ms lead to changes in the in-phase rate of 172, 124, and 310 spikes/sec in two-compartment active, single-compartment active, and single-compartment passive models, respectively.

### Efficiency and reliability

In order to examine the numerical efficiency of the models, we calculated their relative integration times (Fig 4A). The two-compartment active IF model can be computed roughly three times faster than the active Na model, because of its much smaller number of active currents. The allowable time step Δt for the two-compartment active IF model, below which numerical calculation was reliable, was ten times larger than that for the active Na model. For Δt ≥ 3 μs, numerical results show oscillations at the spike initiation leading to a computational unreliability (Fig 4B; see Materials and Methods for the definition of the reliability).

Numerical integration of the single-compartment active IF model was about 10% faster than the two-compartment model (Fig 4A). A substantially larger time step Δt than the two-compartment model was accepted, because the single-compartment model lacks the node which is small and more vulnerable to spike-induced currents. The maximum allowable time step was about 15 μs. Exceeding this criterion resulted in unreliable calculation of spike waveforms (Fig 4C). The computational efficiency of the passive IF model was roughly three times better than the other two active models and the non-spiking model (Fig 4A). However, the maximum time step, which was determined by the reliability of the numerical calculation (Fig 4D), was similar to that of the single-compartment active IF model.

### Comparison of three reduced models

Starting from the active Na model (Fig 1A), we constructed three reduced models step-by-step. The two-compartment active IF model (Fig 1E_1_) and the single-compartment active IF model (Fig 1F_1_) showed similar physiological properties to previous experimental results. The single-compartment active IF model was slightly more robust to parameter changes, and allowed five times larger time step with a subtly better computational speed. Our results are summarized in Table 3.

**Table 3:** Summary of tested physiological reproducibility and computational performance of the three reduced models.

Property	2-comp active IF	1-comp active IF	1-comp passive IF
Simulated waveform	OK (Figs 1E_2_ and 2B)	OK (Figs 1F_2_ and 2C)	OK (Figs 1G_2_ and 2D)
Phasic spiking	Yes (Fig 1E_3_)	Yes (Fig 1F_3_)	No (Fig 1G_3_)
Phase coding	OK (Fig 3A_1_)	OK (Fig 3B_1_)	Too steep (Fig 3C_1_)
Threshold change	Relatively robust (Fig 3A_2_)	Relatively robust (Fig 3B_2_)	Relatively sensitive (Fig 3C_2_)
Refractory period change	Relatively robust (Fig 3A_3_)	Most robust (Fig 3B_3_)	Relatively sensitive (Fig 3C_3_)
Maximum allowable time step (Fig 4A)	2 μs	15 μs	15 μs

Corresponding figures are indicated in brackets.

The active K_LVA_ conductance was found to reduce low frequency components of the input [28,32] and to promote coincidence detection [16,40]. Since the single-compartment passive IF model (Fig 1G_1_) lacks this conductance, its response properties differed substantially from the two active models. Changes in inputs (Fig 3C_1_) and parameters (Fig 3C_2_ and 3C_3_) more severely affect the coding property of the passive IF model.

In conclusion, we suggest the single-compartment active IF model as the minimal conductance-based model that satisfies fundamental electrophysiological properties of auditory coincidence detectors and that showed the best robustness to threshold parameter changes.

## Discussion

### Technical limitations

In any neuronal modeling, there is always a compromise between specification and abstraction [26]. In this study we focused on finding the minimal configuration required for simulating known physiological functions of the owl's coincidence detector neuron. We introduced three different IF models that were reduced from the HH-type conductance based model. Despite its name "integrate", an IF model with a very short time constant behaves not like an integrator but rather like a coincidence detector [46]. As far as we tested, the single-compartment active IF model showed the best results in terms of physiological reproducibility and computational performance. The active K_LVA_ conductance in our models contributed in stabilizing the model responses to parameter changes.

Because we replaced Na conductance with a thresholding unit, the reduced IF models do not show Na-related phenomena such as an improvement of small signal detection [16,27]. Reducing the number of parameters, however, may allow for more rigorous mathematical treatments (e.g., analysis on IF models receiving periodic inputs [24,47–50]; two-dimensional phase-plane analysis [19,27]). Applying these mathematical techniques to our simplified models will be a future research subject.

Implementation of a neuronal model always requires a careful selection of the time step Δt, because it affects the numerical accuracy. Reduced models generally allow larger time steps (Fig 4A). The single-compartment active IF model was compatible with a maximum time step of 15 μs, when the explicit Euler method was used. Our previous study suggested that, to quantify phase-locking, the sampling frequency should be at least 10 (ideally 20) times larger than the locking frequency [51]. Therefore, a time step of 10–15 μs would be the lowest precision allowed for systems like the owls' auditory brainstem, which shows phase-locking up to about 8 kHz [36]. Nevertheless, if a larger time step is required, an implicit or exponential method may need to be considered [52].

### Possible applications

Our simulations showed that it is not always necessary to include all active ionic currents (such as fast Na and delayed rectifier K) to properly simulate auditory coincidence detector neurons. This suggests that functional roles of various ion channels (e.g., [53]) may be separately investigated by restricting the number of ion channels combined with the IF-based model. Further physiological investigations would be necessary for evaluating the applicability of the reduced models for these purposes.

Because of the computational portability, IF models were often used for simulating a network of neurons (e.g., [54–56]). In the auditory brainstem of mammals and birds, a huge extracellular field potential called the neurophonic is observed [57,58]. The amplitude of the neurophonic often exceeds 1 mV, but its functional significance (if any) is mostly unknown. Our reduced models may be useful for examining these collective effects of a large number of brainstem neurons. For such cases, the two-compartment active IF model, which showed less computational performance than the single-compartment model, might also be of some use, because the shape of a neuron affects the formation of the extracellular potential [58,59].

Both in birds and mammals, the auditory brainstem response (ABR) has been widely used as non-invasive diagnostic and research tools. Various auditory stations differentially contribute to the peaks of ABR waveforms [60,61], but how the underlying neural activity is related to the formation of ABR remains to be investigated. Future studies using a network of simplified brainstem neuron models may contribute in identifying the mechanisms of these sound-induced electric responses.

In summary, our results suggest that a single-compartment active IF model with relatively small number of parameters can simulate auditory coincidence detectors that sense submillisecond differences of binaural inputs. Our reduced models would serve as a useful tool for a theoretical investigation on the collective functions of these neurons.
